# The Impact and Safety of GLP‐1 Agents and Breast Cancer

**DOI:** 10.1002/cam4.70932

**Published:** 2025-06-24

**Authors:** Kayla Parsons, Mateo Montalvo, Neal Fischbach, Melissa Taylor, Salome Alfaro, Maryam Lustberg

**Affiliations:** ^1^ Yale University New Haven Connecticut USA; ^2^ Bridgeport Hospital Bridgeport Connecticut USA; ^3^ Yale Medicine New Haven Connecticut USA

**Keywords:** bariatric surgery, behavioral modification, breast cancer, GLP‐1 receptor agonists, obesity, pharmacological intervention, sarcopenic obesity, weight management

## Abstract

**Introduction:**

Obesity and breast cancer rates are increasing globally, with obesity prevalence more than doubling since 1990. By 2022, 44% of women were overweight, and 18% were obese. Breast cancer remains the most common malignancy among women, with 2.2 million new cases in 2020. A significant proportion of breast cancer patients are overweight or obese at diagnosis, which is associated with higher recurrence and mortality rates. Recently, GLP‐1 receptor agonists (GLP‐1 RAs) have emerged as remarkably effective weight loss drugs. Understanding the relationship between obesity, breast cancer, and weight loss is crucial for improving patient outcomes.

**Methodology:**

A comprehensive review of literature from 1996 to 2024 was conducted using databases such as PubMed, Medline, and Web of Science. Studies included epidemiological data on obesity and breast cancer incidence, systematic reviews, meta‐analyses, and clinical trials on weight management interventions (behavioral modification, bariatric surgery, and pharmacological treatments) for breast cancer patients. Preclinical studies examining the biological mechanisms linking obesity and breast cancer progression were also reviewed.

**Results:**

Epidemiological studies consistently show that overweight and obese post‐menopausal women have an increased risk of developing breast cancer. Obesity at diagnosis is linked to worse outcomes, including higher disease recurrence, breast cancer‐specific mortality, and all‐cause mortality. Weight gain during treatment, particularly with chemotherapy, is common and often leads to sarcopenic obesity. Behavioral interventions have shown modest weight loss but are difficult to maintain. Bariatric surgery reduces the risk of developing breast cancer but lacks data on its impact on tumor characteristics and recurrence. GLP‐1 receptor agonists like semaglutide and tirzepatide have demonstrated significant weight loss in non‐cancer populations, but their safety and efficacy in breast cancer patients are not well established.

**Discussion:**

The biology underlying obesity's role in breast cancer progression involves complex interactions between adipocytokines, hormones, and inflammatory cytokines. Weight loss interventions have potential benefits, but sustaining weight reduction in breast cancer patients is challenging. The emerging pharmacological treatments, particularly GLP‐1 receptor agonists, show promise for effective weight management but require further investigation to confirm their safety and impact on breast cancer outcomes.

**Conclusion:**

Addressing obesity in breast cancer patients is critical for improving prognosis and quality of life. While current data do not suggest adverse safety signals with GLP‐1 receptor agonists, more research is needed to fully understand their effects. Effective, safe, and sustainable weight management strategies are urgently needed to support breast cancer patients.

## Graphical abstract

Weight gain is a common side effect of breast cancer treatment and is associated with increased cancer progression and mortality. GLP‐1 receptor agonists are emerging as potential weight loss therapies for patients with breast cancer. There are key questions central to this paper: Are GLP‐1 agents safe? Do they promote weight loss, and do they improve breast cancer outcomes?.

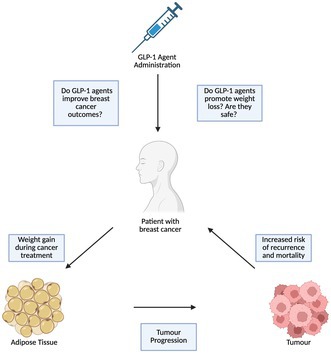

## 1 Background

Obesity and breast cancer rates are increasing worldwide. The global prevalence of obesity in adults has more than doubled since 1990 [1]. In 2022, around 44% of women were overweight, and 18% were obese [1]. Breast cancer is the most common malignancy, and its global incidence in women was 2.2 million cases in 2020 [2]. Obesity is associated with a higher risk of developing breast cancer [3] and between 50% and 90% of women with breast cancer gain weight during treatment [4, 5]. There is a strong association between the two diseases, with many epidemiological studies demonstrating that overweight or obese post‐menopausal women have an increased risk of developing breast cancer [4, 6, 7, 8]. Furthermore, women who were obese at diagnosis have an increased risk of disease recurrence [9], breast cancer‐specific mortality [10, 11] and all‐cause mortality [12, 13, 14]. In 2014, a systematic review and meta‐analysis of 82 follow‐up studies on the body mass index (BMI) and survival of women with breast cancer found that obesity was consistently associated with an increased risk of total mortality and breast cancer mortality (41% and 35% respectively) compared with normal‐weight women [15].

Independent of breast cancer mortality, obesity increases the risk of co‐morbid conditions, including cardiovascular disease (the most common cause of non‐cancer‐related death in breast cancer patients) and diabetes. Additionally, obesity increases surgical complications (e.g., surgical site infections and wound disruption) [16].

Weight gain after receiving a breast cancer diagnosis is common in both pre‐ and post‐menopausal women, with post‐diagnosis weight increases ranging on average up to 5.0 kg or greater [17, 18]. Both retrospective [19] and prospective studies [17, 20, 21, 22, 23, 24] have demonstrated that weight gain is associated with a variety of cancer therapies, particularly chemotherapy [4]. Post‐treatment weight gain alters body composition through gains in fat mass; specifically, central fat, while muscle mass remains unchanged or may decline [4]. This type of weight gain is known as sarcopenic obesity, which has adverse impacts on bodily functions, such as reduced muscular strength and mobility [4].

The biology underlying the precise role of obesity in promoting breast cancer progression is not clearly defined [25]. Many energy‐balance‐related factors such as leptin, insulin‐like growth factor‐1, and sirtuins are known to influence tumor progression [25]. The interaction of obesity‐related hormones, adipocytokines, and inflammatory cytokines is hypothesized to stimulate antiapoptotic genes and impact cell migration, proliferation, and angiogenesis in a pro‐oncogenic fashion [25, 26]. As an example, obese postmenopausal women produce higher levels of estradiol through the aromatization of androgens in adipose tissues [27], and obesity is associated with higher levels of insulin [28]. Both elevated estradiol levels and hyperinsulinemia are correlated with a worse prognosis in breast cancer [15]. Prospective trials evaluating weight loss and lifestyle interventions have yet to convincingly demonstrate a causal relationship between weight loss and improved breast cancer outcomes, though these trials have been limited by low rates of achieving target weight loss. Subset analyses clearly suggest that individuals who lose weight have improved outcomes, but overall results have been inconclusive [29].

## 2 Existing Treatments for Weight Reduction

There are three broad approaches used for weight reduction: behavioral modification, bariatric surgery, and pharmacological intervention. The largest body of data demonstrates that behavioral modification, including dietary and exercise recommendations, may improve the quality of life for breast cancer patients [30]. A systematic review of 17 reviews conducted in 2022 found that a majority of the studies (twelve) reported modest weight loss between 1.36 kg (95% CI: −2.51 to −0.21) to 3.8 kg (95% CI: −5.6 to −1.9) for breast cancer patients participating in multicomponent behavioral intervention studies [31, 32]. However, these results came from intensive studies that are challenging to replicate in a real‐world setting, and many breast cancer survivors struggle with sustainable weight loss [33, 34].

Bariatric surgery is another strategy for a durable treatment for obesity and has been associated with a decreased risk of developing breast cancer [35, 36, 37, 38, 39, 40]. However, few studies have researched the impact of bariatric surgery on breast cancer tumor characteristics like hormone receptor status, and even fewer have evaluated the impact of bariatric surgery on breast cancer recurrence [35, 40].

Obese and overweight adults who have difficulty achieving weight loss with lifestyle interventions alone generally seek out long‐term pharmacological therapy [41]. Many medications developed to treat obesity have also been used to treat obesity in the breast cancer population. Pharmacological intervention decreases the risk of obesity‐related complications such as cardiovascular disease and hypertension [41, 42]. Prior to 2023, the FDA approved weight‐loss drugs including phentermine, topiramate, and lorcaserin [43]. These medications should be combined with lifestyle modifications for full benefits [43]. Unfortunately, these medications have modest activity and significant potential for toxicity [44]. Compounding the difficulty with pharmacotherapy for obesity in patients with breast cancer, recent findings demonstrated that breast cancer survivors on aromatase inhibitors and anti‐obesity medications had poorer weight loss outcomes than patients on anti‐obesity medications without breast cancer and not on aromatase inhibitors [45].

## 3 GLP‐1 Agents and Weight Loss

The landscape for weight loss changed dramatically with the development of glucagon‐like‐peptide‐1 (GLP‐1) receptor agonists. GLP‐1 receptor agonists are incretin mimetic drugs that facilitate fasting and postprandial glycemic control by increasing glucose‐dependent insulin secretion, reducing glucagon release, suppressing hepatic gluconeogenesis, and slowing gastric emptying [46]. These agents were initially developed for managing type 2 diabetes (T2DM) but now are proven to be remarkably effective weight loss drugs (Table 1) [47, 48, 49, 50].

**TABLE 1:** GLP‐1 RA medications.

Drug	Mechanism of action	Duration of action	Indications
Exenatide, lixisenatide	GLP‐1 RA	Short‐acting	Diabetes type 2
Exenatide extended‐release, dulaglutide, albiglutide	GLP‐1 RA	Long‐acting	Diabetes type 2
Liraglutide, semaglutide	GLP‐1 RA	Long‐acting	Diabetes type 2, weight management
Tirzepatide	GLP‐1/GIP RA	Long‐acting	Diabetes type 2, weight management

Abbreviations: GIP, glucose‐dependent insulinotropic polypeptide; GLP‐1, glucagon‐like peptide‐1; RA, receptor agonist.

Semaglutide was the first GLP‐1 agonist FDA‐approved for chronic weight management [51]. In the FDA registration trial, 1961 patients with BMI > 30 or BMI > 27 with a comorbid condition were randomized 2:1 to semaglutide versus placebo, both with lifestyle interventions [52]. The dose of semaglutide was initiated at 0.25 mg and escalated to a target dose of 2.4 mg weekly [52]. The study demonstrated a remarkable 14.9% reduction in body weight in the semaglutide arm and a 2.7% reduction in the placebo arm, with an estimated treatment difference of −12.4% points (95% CI: −13.4 to −11.5; *p* < 0.001) [52]. It is important to note that breast cancer patients were excluded from this trial.

Semaglutide demonstrated similar side effect profiles to other GLP‐1 receptor agonists, including gastrointestinal (GI) side effects such as nausea, diarrhea, and vomiting [53]. Other side effects consist of fatigue, dizziness, anxiety, gallbladder disease, and pancreatitis [53]. There is conflicting data regarding the relation of semaglutide to acute kidney injury and thyroid malignancies [54]. However, semaglutide is contraindicated in patients with a family or personal history of medullary thyroid cancer and in patients with multiple endocrine neoplasia type 2 until new data is available.

Tirzepatide was initially approved by the FDA in 2022 as a once‐weekly subcutaneous injectable peptide for T2DM [55]. Phase 2 trials in people with T2DM found tirzepatide was associated with significant weight loss [55]. Subsequently, a phase 3 randomized, controlled trial analyzed 2539 adults with a BMI of 30 or more or 27 or more with at least one weight‐related complication (excluding diabetes) [55]. Participants received tirzepatide once weekly on an escalating protocol to a target dose of 15 mg or placebo for 72 weeks [55]. The study found that adults who received 5, 10, and 15 mg doses of tirzepatide experienced average weight reductions of 15% (95% CI: −15.9 to 14.2), 19.5% (95% CI: −20.4 to −18.5) and 20.9% (95% CI: −21.8 to −19.9) of initial body weight, respectively [55]. The most frequently reported adverse events were nausea, diarrhea, and constipation [55]. Similar to semaglutide, patients with a history of breast cancer were not included in the trial.

Orforglipron is a once‐daily oral nonpeptide GLP‐1 receptor agonist that is currently undergoing development to manage weight and treat T2DM [56]. Unlike peptide GLP‐1 agonists, orforglipron is orally bioavailable and has a shorter half‐life. In a phase 2, randomized and placebo‐controlled trial, a total of 272 participants underwent randomization [56]. The study found that the use of orforglipron led to a consistent reduction in body weight that varied depending on the dosage [56]. Patients who received orforglipron lost around 9% to 13% of their body weight versus the placebo group who lost 2% [56]. The most common adverse effects were GI events including nausea, constipation, vomiting, diarrhea, and eructation [56]. However, breast cancer patients were also not included in this study.

Liraglutide is a well‐tolerated GLP‐1 agonist approved for use in adults with T2DM that is associated with improved glycemic control and reduction in weight [57]. Seven phase 3, randomized trials analyzed the efficacy and safety of liraglutide in T2DM and found that many subjects experienced > 5% weight loss [57]. Breast cancer patients were not included in these study populations. Similar to other GLP‐1 agonists, GI side effects, especially nausea, headaches, and dizziness, are the most common toxicities, though generally mild [57].

Finally, exenatide is a synthetic form of exendin‐4 approved for use in patients with T2DM who are taking metformin [58]. A 52‐week phase 3 trial that compared twice‐daily exenatide and biphasic insulin aspart found that exenatide had a similar glycemic effect to premixed insulin and resulted in significant weight loss. On average, patients treated with exenatide lost 5.4 kg more weight compared to those treated with biphasic insulin aspart [58]. Another phase 3 study in patients with T2DM found similar results, with exenatide significantly reducing patients' HbA_1c_ and promoting weight loss [59]. Common side effects of exenatide include nausea, headaches, diarrhea, and hypoglycemia [60], however, more serious side effects include pancreatitis and kidney problems [61]. Currently, weight loss remains an off‐label indication for exenatide.

## 4 Controversies With Safety of GLP‐1 Agents

Federal Drug Administration (FDA) Adverse Event Reporting System (FAERS) database ranked the top six adverse events for GLP‐1 agents as nausea, vomiting, diarrhea, upper abdominal pain, constipation, and less frequently, pancreatitis [62]—a potentially fatal condition [63]. Early clinical trials reported an increased risk of developing acute pancreatitis with GLP‐1 treatments compared to placebo or other active comparators [64]. However, several studies and meta‐analyses [65, 66, 67, 68, 69, 70, 71] demonstrated no increased risk of pancreatitis with GLP‐1 RAs, including a meta‐analysis with over 55 randomized controlled trials [65, 72]. Ultimately, there is no definitive causal relationship established between GLP‐1 RA use and pancreatitis [65]. Nevertheless, when prescribing GLP‐1 agents, physicians should consider specific risk factors such as T2D and hypertriglyceridemia, which may contribute to the development of pancreatitis [65].

Moreover, there are concerns about the safety of GLP‐1 agents in terms of thyroid cancer that are mainly based on rodent studies [73, 74, 75]. These studies suggest that GLP‐1 RAs may trigger parafollicular C‐cell proliferation, potentially influencing the risk of new‐onset medullary thyroid neoplasms [76, 77]. However, observations in clinical studies have not confirmed this cancerogenic effect of exenatide or liraglutide except at doses eight times higher than the greatest dose approved for liraglutide in humans [78], suggesting a potential dose‐dependent effect on thyroid cell proliferation, particularly in dysplastic or premalignant lesions [76]. Additionally, a large multisite study and cohort study found no association between GLP‐1 RA use and an increased risk of thyroid cancer after a follow‐up between 1.8 and 3.9 years [79, 80, 81]. Further studies with long‐term follow‐up are needed to elucidate the role of GLP‐1 RAs in thyroid and medullary thyroid carcinomas.

## 5 GLP‐1 Agents and Breast Cancer in Preclinical Studies

In preclinical models, exendin‐4 was found to inhibit the proliferation of MCF‐7 breast cancer cells and enhance apoptosis via the modulation of apoptosis‐related genes [82]. MDA‐MB‐468 cells were injected into mice, and they received subcutaneous osmotic pumps or exendin‐4 with varying concentrations, and researchers found that exendin‐4 reduced the growth of breast cancer cells in vivo [83]. Tumor weight was found to decrease after implanting exendin‐4 subcutaneously, and when exendin‐4 was added to MCF‐7, MDA‐MB‐231, MDA‐MB‐468, HB2, and HEK‐293 cells, there was a 50% growth inhibition of breast cancer cells [83].

Another preclinical study found that treatment with 0.1 to 10 nM exendin‐4 significantly decreased the proliferation of MCF‐7, MDA‐MB‐231, and KPL‐1 cells in a dose‐dependent manner. However, treatment with 10 nM of exendin‐4 in these cell lines did not induce apoptosis. Because GLP‐1R gene expression was the most abundant in MCF‐7 cells, the researchers used this cell line for the following experiments. They discovered that the GLP‐1R antagonist exendin completely reversed the antiproliferative effect of exendin‐4 and that GLP‐1R knockdown eliminated the reduction in MCF‐7 cell numbers by exendin‐4, suggesting that exendin‐4 reduces breast cancer cell numbers through GLP‐1R activation. In athymic mice, tumor size decreased after exendin‐4 treatment to almost half the size of the tumors of the control. Further, Ki67—a marker of cell proliferation and cell progression—significantly decreased following exendin‐4 treatment as well, but when mice were treated with exendin‐4 and exendin, the reduction in Ki67 mRNA was critically enhanced with expression levels similar to control levels, further suggesting that exendin‐4 attenuates breast cancer growth via GLP‐1R and through inhibition of cell proliferation in vivo. Finally, exendin‐4 significantly inhibited NF‐*κ*B (p65) translocation into the nucleus in MCF‐7 cells, a mechanism that was eliminated by the GLP‐1R antagonist exendin, proving again that this interaction occurs via GLP‐1R activation. These findings propose that exendin‐4 could attenuate breast cancer cell proliferation by inhibiting the NF‐*κ*B activation which reduces the expression of its target genes [84].

Building off this study, the researchers examined combined therapy with exendin‐4 and metformin—a diabetes agent generally used as first‐line therapy for T2D with a glucose‐lowering effect as well as an anti‐cancer effect—in both cell culture assays and in vivo with athymic mice. There was a significant reduction in tumor volumes observed only in the metformin group and not the exendin‐4 group in athymic mouse tumor models. However, with combined therapy, the tumors had the smallest weight. Lastly, immunofluorescence analysis demonstrated that exendin‐4, metformin, and combined therapy significantly decreased Ki67‐positive proliferative cells [85]. This body of data suggests that there may be synergistic activity between GLP‐1 agonist and other metabolic pathway therapies.

In the early stages of breast cancer development, mechanisms like DNA methylation induce epigenetic modifications. In TNBC, methylation of certain promoter regions of key genes can alter gene expression, resulting in resistance to hormonal treatment and the promotion of more aggressive tumor characteristics. Chequin et al. examined liraglutide's potential in modulating epigenetic mechanisms in a preclinical study. Liraglutide reduced tumor cell growth and inhibited colony formation in all cell lines by almost twofold compared with the control group. It also reduced cell migration of MDA‐MB‐231 and MDA‐MB‐436 cells and acted as a DNA methylation inhibitor along the *ADAM33* gene promoter in vitro. Moreover, liraglutide decreased DNMT activity and global DNA methylation in MCF7, MDA‐MB‐231, and MDA‐MB‐436 cell lines. Specifically, liraglutide reduced DNMT activity in the MDA‐MB‐436 cell line to 60% ± 1.5%, indicating that this treatment inhibited enzyme activity more effectively than 5‐Aza‐dc and exenatide. In vivo, treatment with a daily subcutaneous injection of 19 mg/kg (correlated to the human dose) reduced tumor volumes similar to methotrexate (MTX) and paclitaxel (PAC). Combined treatment with liraglutide and PAC yielded a reduction in tumor size of 78% when compared with the control group and 39% compared with only PAC‐treated animals. Similar to the results in the breast cancer cell lines, the liraglutide treatment produced significant demethylation in CDH1 and ADAM33 promoter regions. Specifically, in the ADAM33 promoter region, there was a significant reduction in the methylation profile of CpG islands close to 55%. These results prove that liraglutide treatment reached the tumors and induced demethylation. This increase in gene expression may lead to potentially improved tumor prognosis [86].

## 6 GLP‐1 Agents and Breast Cancer in Clinical Studies

The largest analysis of the impact of GLP‐1RAs and cancer risk is a retrospective cohort study of over 1.6 million T2D patients without prior diagnoses of obesity‐associated cancers (OACs) treated with GLP‐1 RAs in addition to other hypoglycemic agents from March 2005 to November 2018. OACs refer to cancers where excess body fat is associated with an increased risk of developing cancer and a worse prognosis [87]. Risk of 13 OACs—esophageal, breast, colorectal, endometrial, gallbladder, stomach, kidney, ovarian, pancreatic, and thyroid cancer as well as hepatocellular carcinoma, meningioma, and multiple myeloma—was assessed and correlated with hypoglycemic therapy. After a 15‐year follow‐up, the study found a significant reduction in several OACs. However, there was no association between GLP‐1RAs and reduced risk of postmenopausal breast cancer and no clear difference in breast cancer incidence among postmenopausal women treated with GLP‐1RAs compared to those treated with insulin or metformin [87].

The impact of GLP‐1 agonists on weight change in breast cancer patients has been analyzed in several retrospective studies. One analysis included 75 patients with breast cancer who started treatment with GLP‐1 agonists with follow‐up weight data before and after GLP‐1 agonist therapy [88]. The median weight reported at GLP‐1 agonist initiation therapy was 90 kg (58–151). The median duration of treatment was 20 months (6–111). The mean relative weight change was −5% (SD 6%) at 12 months after GLP‐1 agonist initiation. Another study analyzed the effect of semaglutide, liraglutide, dulaglutide and tirzepatide on weight loss outcomes in breast cancer patients who were taking aromatase inhibitors or SERM [89]. The results showed a mean percentage change in BMI with semaglutide in a −4.34% reduction; with liraglutide −3.51%, dulaglutide −2.33%, and tirzepatide showed a 2.31% change at the 12‐month mark. When compared to weight reductions of −14% with semaglutide, −8.4% with liraglutide, −10% with dulaglutide and −15% with tirzepatide in the general population, the authors concluded that the impact of GLP‐1 agonists may be attenuated in women with breast cancer receiving endocrine therapy [89]. Another study showed that the impact of tirzepatide on weight loss was greater than semaglutide in adults who are overweight or obese, demonstrating different effects in comparison to the breast cancer population [90].

The safety of GLP‐1 agonists for weight loss in patients with a history of breast cancer remains undefined as this group was excluded from the large, randomized trials leading to FDA approval of tirzepatide and semaglutide. However, the incidence of breast cancer in subjects treated with GLP‐1 receptor agonists was reviewed in a systematic review and patient‐level meta‐analysis in 2021. That study found no increased risk of breast cancer among roughly 46,000 GLP‐1 treated patients with roughly 41,000 controls [47]. Similarly, the previously described large retrospective cohort study of patients with T2DM found no increased incidence of breast cancer and decreased incidence in several other OACs. Expanding on this, a separate study using a nationwide multicenter database of electronic health records of over 1.6 million patients with T2D with no prior diagnoses of OACs and who were prescribed GLP‐1 RAs, insulins, or metformin, found no clear association between GLP‐1 RA use and a reduced risk of developing postmenopausal breast cancer [91].

In patients with polycystic ovarian syndrome (PCOS), a retrospective cohort study compared metformin and GLP‐1 RA treatment and found that GLP‐1 RA was associated with a lower risk of incident breast cancer compared to patients taking metformin. Moreover, several other population‐based trials have found no association between the use of GLP‐1 analogs and increased risk of breast cancer (Tables 2 and 3) [47, 92, 93, 94, 95, 96].

**TABLE 2:** GLP‐1 RA and weight outcomes on patients with breast cancer.

Study	Population	Intervention	Duration	Weight outcomes	References
Sukumar et al. (2024)	Patients with breast cancer	GLP‐1 RA	Variable, median 1.2 years (range 0.3–8.1)	Median weight loss −2.1% at 3 months, −2.9% at 6 months and −3.1% at 12 months	[100]
Bhave et al. (2024)	Patients with breast cancer	GLP‐1 RA	Variable, median 20 months (range 6–111)	Mean relative weight change: −5% at 12 months after initiation. −2.8 kg at 6 months, −4.2 kg at 12 months, and −6.2 kg post‐treatment	[104]
Fischbach et al. (2024)	Patients with breast cancer	Semaglutide/tirzepatide	Variable	Mean weight loss: 3.03 kg, BMI reduction of 1.1 kg/m^2^	[98]
Portillo et al. (2024)	Patients with breast cancer on hormonal therapy	Semaglutide, liraglutide, dulaglutide, and tirzepatide	12 months	Mean percentage changes in BMI: Semaglutide: −4.34%, Liraglutide: −3.51%, Dulaglutide: −2.33%, and Tirzepatide: −2.31%	[89]

**TABLE 3:** GLP‐1 RA and breast cancer risk.

Study	Population	Intervention	Duration	Risk of breast cancer	References
Sun et al. (2024)	Adults with and without cancer	GLP‐1 RA	Variable	Reduced risk of breast cancer (OR 0.92, 95% CI 0.88–0.96)	[94]
Levy et al. (2024)	Adults with obesity	GLP‐1 RA	5‐year follow‐up	Reduced risk of breast cancer (HR 0.72, 95% CI 0.64–0.82)	[95]
Piccoli et al. (2021)	Adults with obesity and/or diabetes	GLP‐1 RA	Variable, minimum 24 weeks	No increased risk of breast cancer (RR 0.98, 95% CI 0.76–1.26)	[47]
Hicks et al. (2016)	Women with type 2 diabetes	GLP‐1 vs. DPP‐4 inhibitors	Variable, mean follow‐up 3.5 years	No increased risk of breast cancer compared to DPP‐4 (HR 1.40, 95% CI 0.91–2.16)	[96]

Notably, these pooled analyses included limited patients treated with semaglutide or tirzepatide. One prospective RCT involving 665 patients with T2DM randomized to 2 doses of semaglutide versus physician's choice of additional anti‐diabetic agent (OAD) found no increase in cancer compared to OAD, though follow‐up was only 56 weeks. Further, in a single‐arm clinical trial with the same exposure to semaglutide, there were no significant differences in the development of neoplasms when comparing the semaglutide group to the OAD [97]. One retrospective study analyzed the association of semaglutide and tirzepatide use with weight loss in patients with early breast cancer (stage I‐III). Of the cohort of 5430 patients, 74% of patients received endocrine therapy. The mean weight loss for semaglutide and tirzepatide patients was 3.03 kg (3.9–2.13, *p* < 0.0001) with a BMI reduction of 1.1 kg/m^2^ (0.7–1.5, *p* < 0.0001). The mean maximal weight loss was 8.89 kg (6.71–10.98, *p* < 0.0001) with a BMI reduction of 3.2 kg/m^2^ (2.3–4.2, *p* < 0.0001). The rates of local, locoregional, and distant breast cancer recurrence in patients taking semagutide and tirzepatide compared to untreated patients were not statistically different [98]. Additionally, there is an ongoing single‐arm, phase II, non‐randomized trial for weekly administration of tirzepatide in patients with early‐stage HR+/HER2‐BC with a BMI ≥ 30 kg/m^2^ or ≥ 27 kg/m^2^. The primary outcome of interest is to determine the percentage of patients who achieved a weight loss of more than 5% [99].

The largest study to date that analyzed patterns of GLP‐1 RA use in breast cancer survival was a retrospective study that analyzed roughly 1022 patients with non‐metastatic (DCIS or invasive stage I‐III) at MD Anderson Cancer Center who received at least 3 months of semaglutide and tirzepatide. Among the 389 patients with invasive breast cancer who received GLP‐1 RAs, the median weight loss was −2.1%, −2.9%, and −3.1% at 3, 6, and 12 months, respectively. Multivariate regression analysis found no association between 6‐month weight gain and clinical factors such as type 2 diabetes (DM2), metformin use, endocrine therapy use, clinical stage, and GLP‐1 RA use. However, at 12 months, weight gain was primarily associated with endocrine therapy (tamoxifen or aromatase inhibitor) with patients gaining 2.36 kg on average compared to those not on endocrine therapy, indicating that this treatment could have impacted weight loss for patients on GLP‐1 RAs. In this study, there were no significant differences in disease‐free survival between those who received GLP‐1 RAs and those who did not. In contrast, there was a significant improvement in overall survival (OS) for patients on GLP‐1 RAs compared to the control group; the median OS (0–30.7 years) was not reached for GLP‐1 RA patients, while the control group OS was 24.1 years. The researchers concluded that clinical trials examining the role of GLP‐1 RAs for weight loss in breast cancer survivors are necessary as well as further investigation into the potential biological effects of GLP‐1 RAs on cancer [100].

Additional outcomes, including the pathological complete response (pCR), were studied in a retrospective analysis of 347 patients with early‐stage triple‐negative breast cancer (TNBC) who received KEYNOTE‐522 and GLP‐1 RAs or DPP4i treatment at diagnosis and throughout the neoadjuvant period. The pCR rate for the patients on GLP‐1 RAs or dipeptidyl peptidase 4 inhibitors was 28% compared to 63.66% for the patients on other classes of diabetes medication or no medication. Additionally, there was GLP‐1 RA expression in tumor cells and infiltrating immune cells in human TNBC specimens, suggesting potential immunomodulatory effects that can impact treatment response. Grade, age, BMI and diabetes were key determinants of pCR in multivariate analysis [101].

While these studies report participant demographics such as race, age, and gender, there is a lack of analysis on the influence of socioeconomic status, race/ethnicity, and age on treatment outcomes. Although some articles acknowledge the preponderance of White, female [52, 56] participants as a study limitation, they do not systematically evaluate its impact on treatment efficacy, nor do they propose methodological adjustments for future research. Two studies addressed age‐related limitations. One study noted that the predominance of younger participants limited the generalizability of the findings [49], while another pointed out the large age gap between participants as an area of concern [46]. Addressing these limitations is crucial for enhancing the generalizability of findings or determining the necessity of race‐, gender‐, or age‐specific investigations.

While there are not yet safety signals to suggest a negative impact of GLP‐1 agonists on breast cancer outcomes, there are some preclinical models suggesting a possible deleterious interaction. Liu et al. demonstrated expressions of GLP‐1 receptors in human TNBC cells and tissues from BALB/cfC3H mice bearing 4T1 tumor cell implants and tumor cell growth increased with liraglutide injection [102]. The researchers also demonstrated that in two highly aggressive TNBC cell lines, MDA‐MB‐231 and MDA‐MB‐468, GLP‐1 receptor activation by liraglutide may promote breast cancer cell growth through the NOX4/ROS/VEGF pathway and that liraglutide acted as a tumor promoter at a concentration of 100 nM in vitro [102]. Another study reported that liraglutide can promote the growth of MDA‐MB‐231 cells, though this effect was only seen at high concentrations of liraglutide [103].

Although these preclinical studies highlight the potential risks of GLP‐1 receptor agonists with breast cancer, other reports reach the opposite conclusion. In a preclinical trial, MCF‐7 cell lines were treated with different concentrations of liraglutide [93]. The researchers determined that liraglutide inhibited proliferation and stimulated the apoptosis of MCF‐7 human breast cancer cells [93].

Despite evidence supporting GLP‐1 RAs as effective weight loss drugs, there is limited data on their long‐term effects in breast cancer patients, creating a significant knowledge gap insufficiently addressed in existing literature. While clinical and preclinical studies present conflicting results with some studies suggesting that GLP‐1 RAs have no increased risk of breast cancer and others pointing to potential tumor‐promoting effects, there is a critical need for future prospective trials. Given the widespread use of GLP‐1 agents and ongoing safety concerns, future research needs to study these drugs in breast cancer patients, critically evaluating their impact on cancer risk, recurrence, mortality, and long‐term outcomes for this population. Clinical and preclinical studies demonstrate GLP‐1 RAs as excellent weight loss drugs with therapeutic potential for obesity‐related illness, emphasizing the need for rigorous investigation to ensure safe clinical use in breast oncology.

## 7 Conclusion

GLP‐1 agonists, in particular, semaglutide and tirzepatide are remarkably effective weight loss drugs that have demonstrated a myriad of positive health benefits in obese and overweight patients, including, most notably, improved overall survival. As weight gain is a significant problem for women with breast cancer, particularly those receiving chemotherapy and endocrine therapy, safe and effective weight loss drugs for this population are urgently needed. The limited available data to date do not suggest an adverse safety signal in patients with breast cancer. However, additional research is needed to fully evaluate the impact of GLP‐1 agents on weight loss, metabolic profiles, and disease outcomes in patients with breast cancer.

## Author Contributions

**Kayla Parsons:** writing – original draft (lead), writing – review and editing (equal). **Mateo Montalvo:** writing – original draft (supporting), writing – review and editing (equal). **Neal Fischbach:** writing – original draft (equal), writing – review and editing (equal). **Melissa Taylor:** writing – original draft (equal), writing – review and editing (equal). **Maryam Lustberg:** supervision (lead), writing – original draft (equal), writing – review and editing (equal). **Salome Alfaro:** validation (equal), writing – review and editing (equal).

## Conflicts of Interest

Maryam Lustberg is a consultant to Novartis, Lilly, Pfizer, Gilead, and Menarini. The remaining authors declare not conflicts of interest.

## Data Availability

This is a review article and does not include any original data.
